# Comparative Genomics of Isolates of a *Pseudomonas aeruginosa* Epidemic Strain Associated with Chronic Lung Infections of Cystic Fibrosis Patients

**DOI:** 10.1371/journal.pone.0087611

**Published:** 2014-02-05

**Authors:** Julie Jeukens, Brian Boyle, Irena Kukavica-Ibrulj, Myriam M. Ouellet, Shawn D. Aaron, Steve J. Charette, Joanne L. Fothergill, Nicholas P. Tucker, Craig Winstanley, Roger C. Levesque

**Affiliations:** 1 Institute for integrative and systems biology (IBIS), University Laval, Quebec City, Quebec, Canada; 2 Centre de recherche de l’Institut universitaire de cardiologie et de pneumologie de Québec, Quebec City, Quebec, Canada; 3 Ottawa Hospital Research Institute, Ottawa, Ontario, Canada; 4 Institute of Infection and Global Health, University of Liverpool, Liverpool, United Kingdom; 5 Strathclyde Institute of Pharmacy and Biomedical Sciences, University of Strathclyde, Glasgow, United Kingdom; UC Berkeley, United States of America

## Abstract

*Pseudomonas aeruginosa* is the main cause of fatal chronic lung infections among individuals suffering from cystic fibrosis (CF). During the past 15 years, particularly aggressive strains transmitted among CF patients have been identified, initially in Europe and more recently in Canada. The aim of this study was to generate high-quality genome sequences for 7 isolates of the Liverpool epidemic strain (LES) from the United Kingdom and Canada representing different virulence characteristics in order to: (1) associate comparative genomics results with virulence factor variability and (2) identify genomic and/or phenotypic divergence between the two geographical locations. We performed phenotypic characterization of pyoverdine, pyocyanin, motility, biofilm formation, and proteolytic activity. We also assessed the degree of virulence using the *Dictyostelium discoideum* amoeba model. Comparative genomics analysis revealed at least one large deletion (40–50 kb) in 6 out of the 7 isolates compared to the reference genome of LESB58. These deletions correspond to prophages, which are known to increase the competitiveness of LESB58 in chronic lung infection. We also identified 308 non-synonymous polymorphisms, of which 28 were associated with virulence determinants and 52 with regulatory proteins. At the phenotypic level, isolates showed extensive variability in production of pyocyanin, pyoverdine, proteases and biofilm as well as in swimming motility, while being predominantly avirulent in the amoeba model. Isolates from the two continents were phylogenetically and phenotypically undistinguishable. Most regulatory mutations were isolate-specific and 29% of them were predicted to have high functional impact. Therefore, polymorphism in regulatory genes is likely to be an important basis for phenotypic diversity among LES isolates, which in turn might contribute to this strain’s adaptability to varying conditions in the CF lung.

## Introduction

*Pseudomonas aeruginosa* is a ubiquitous Gram-negative bacterium that can be found in soil, water and numerous host organisms. In addition to being a leading cause of multidrug-resistant nosocomial infections, this opportunistic pathogen is also the main cause of chronic lung infections among individuals suffering from cystic fibrosis (CF) [1]. In fact, once *P. aeruginosa* has established itself in the CF lung, it is likely to persist for the remainder of the patient’s life and contribute to the decline of pulmonary function [2], [3]. Up until the early nineties, it was thought that CF patients acquired unique strains from the environment. However, in 1996, a strain that could transmit itself among patients was identified and termed the Liverpool epidemic strain, or LES [4]. Since then, LES-like strains have been identified elsewhere in the world, including in Canada [5]. These transmissible strains are associated with increased resistance to antibiotics [6], can cause superinfection of patients previously chronically infected with a different strain and are generally associated with a worse prognosis for patients; hence their discovery has dramatically influenced infection control policies worldwide (reviewed in [7]).

*P. aeruginosa*’s incredible metabolic versatility is generally attributed to genomic diversity [8]. During chronic lung infection, it adapts to CF airways through adaptive and loss-of-function mutations in virulence genes, which are essential for the onset of infection [9]. Genome sequencing of isolate LESB58 revealed that, in addition to a core genome, similar to other reference strains, it carried genomic islands and prophages that are important for *in vivo* competitiveness during chronic lung infection [10]. Still, isolates that do not carry one or more of these regions are common and prophage loss through the course of infection was proposed as an adaptive process, but the exact role played by prophages and the impact of their absence on the host are not understood [11], [12]. Better understanding of *P. aeruginosa* epidemic strain diversity and evolution in the lung environment is critical to achieve more targeted therapy of CF chronic lung infections.

This study focusses on seven isolates of the *P. aeruginosa* LES: LES400, LES431 and LESB65, which were isolated in the UK and exhibit varying levels of virulence in a murine respiratory infection model [13], and four isolates of strain type A, which were isolated in Ontario, Canada and were identified as the same clone type as the LES [5]. To date, this is the only reported case of transcontinental transmission of a *P. aeruginosa* strain in the context of CF lung infections. Previous studies have demonstrated extensive phenotypic variability amongst LES isolates [12], [13], [14], [15]. The main goal of this study was to sequence and annotate the genomes of seven isolates of the LES representing different virulence characteristics and geographic sources in order to associate comparative genomics results with virulence factor variability. We also sought genomic and/or phenotypic differences between the two geographical locations. To this end, we performed *in vitro* phenotypic characterization of motility, biofilm formation, proteolytic activity and exotoxin secretion. We also assessed the degree of virulence using the *Dictyostelium discoideum* amoeba as a host model. This work represents the first detailed comparison of transmissible isolates of the same strain from two different continents.

## Methods

### Ethics Statement

The UK isolates were obtained for diagnostic purposes and are from an existing archived collection. Ethical approval was obtained through local National Research Ethics Service committees and all samples were anonymized. Isolates from Canada were also obtained from an existing collection that was collected for the transmissible strains of *P. aeruginosa* study [5]. IRB approval was given by the Ottawa Hospital Research Institute to use the samples, and all samples were anonymized and identified only by study subject number.

### Sample Preparation

Three UK isolates of the Liverpool epidemic strain (LES) were included in this study: LES400, a chronic infection CF isolate (1998); LES431, isolated from a non-CF parent of a CF patient (2000); and LESB65, a chronic infection CF isolate (2003) [13], [15]. We also included 4 CF isolates of strain type A from Ontario, Canada: LESlike1 (patient 01-022-1, Ottawa, 2005); LESlike4 (patient 03-019-10, Toronto, 2005); LESlike5 (patient 03-054-2, Toronto, 2007) and LESlike7 (patient 05-009-2, Hamilton, 2006) [5]. Genomic DNA was isolated from overnight cultures using the DNeasy Blood and Tissue Kit (QIAGEN).

### Genome Sequencing and Assembly

A detailed description of our approach for genome sequencing, assembly and finishing was previously published [16], [17], [18]. Briefly, whole-genome shotgun DNA sequencing was performed using the Roche 454 pyrosequencing method on the Genome Sequencer FLX system with the Titanium (UK isolates) or Plus chemistry (Canadian isolates) at the Plateforme d’analyses génomiques of the Institute for integrative and systems biology (IBIS, University Laval). Shotgun libraries of the UK strains were sequenced individually on a quarter (LES400, LESB65) or a half plate (LES431), while libraries of the Canadian strains were barcoded and combined on a full plate. Sequence data was analyzed using the gsAssembler module of Newbler v2.5.3 to generate *de novo* contigs (default parameters, options: extend low depth overlaps, reads limited to one contig, complete consed folder), which were aligned against *P. aeruginosa* LESB58 reference genome using BLAST (bl2seq) [19]. Genome finishing was performed using Consed v. 20 [20]. Specific primers were designed inside flanking contigs of all remaining gaps for PCR amplification and Sanger sequencing (primers in Table S1). Automated annotation was performed with xbase [21] and the NCBI Prokaryotic Genome Annotation Pipeline (PGAP) using *P. aeruginosa* LESB58 as a reference genome [10].

### Comparative Genomics

Large insertions, deletions and rearrangements were identified using the Artemis Comparison Tool [22] and the CGView Comparison Tool [23]. We used Panseq analysis to identify core and accessory genome fragments [24]. CLC Genomics Workbench (CLCbio) was used to identify single nucleotide polymorphisms (SNPs) and deletion/insertions polymorphisms (DIPs). Comparative genomics data handling was done using R (r-project, version 2.15.1). Laboratory strain PAO1 was included in comparative genomics analyses as an outgroup, for comparison purposes.

### Strain Phenotyping

As LES isolates grow more slowly compared to laboratory stain PAO1, incubation for bacterial growth on agar media was extended to 48 h instead of 24 h for all protocols cited below. Strain PAO1 was included in phenotypic tests for control purposes. Isolates were inoculated on sheep blood agar to visualize hemolytic activity, identified as a clear zone. Hydrogen peroxide (H_2_O_2_) susceptibility was also assessed [25]. Swimming, swarming and twitching motility were assayed as previously described [26]. Biofilm formation was measured with a 96-well microtiter plate assay according to Carter *et al.* [13]. Pyocyanin production was measured according to Fothergill *et al.* [14] and pyoverdine production was assessed visually under UV light using Pseudomonas Agar F (Difco). Proteolytic activity of culture supernatants was measured using BHI agar plates containing 1% (wt/vol) skim milk [27] and elastolytic activity was determined with the elastin-congo red assay (Sigma-Aldrich) [28]. Additionally, isolates LES431 and LESB58 were compared in a BIOLOG Phenotypic Microarray experiment [29].

### Amoeba Predation Assay

The predation assay was used to determine the bacterial capacity to resist to amoeba grazing. The amoeba was demonstrated to be an effective and simple model system to assess pathogenicity and virulence of *P. aeruginosa* strains [30]. A liquid pre-culture of *P. aeruginosa* strains was done in LB medium. This pre-culture was incubated overnight at 37°C with shaking at 200 rpm. *D. discoideum* amoebae (DH1-10) were grown in HL5 medium (14.3 g/L of bactopeptone, 7.15 g/L of yeast extract, 18 g/L of D-(+)-monohydrate maltose, 0.641 g/L of Na_2_HPO_4_•2H_2_O, and 0.490 g/L of KH_2_PO_4_) [31] with 15 µg/mL of tetracycline. The confluence of amoebae was about 60% on the day of the experiment. A volume of 300 µL of bacterial culture was spread out on a Petri dish containing SM 1/10 agar (1 g/L of bactopeptone, 0.1 g/L of yeast extract, 0.22 g/L of KH_2_PO_4_, 0.1 g/L of K_2_HPO_4_, 0.1 g/L of MgSO_4_•7H_2_O, 2 g/L of bactoagar, and 1 g/L of glucose) to obtain a uniform lawn [32]. Bacteria were then dried in a laminar flow hood, and 5 µl drops of amoebae having concentrations of 50 000, 5 000, 500, 5 and 0 cells/5 µL were deposited on the bacterial lawn. After drops were dried, Petri dishes were incubated at 21°C for 7 days and phagocytic plaques in bacterial lawns due to amoeba growth were monitored.

## Results

### Genome Sequencing and Assembly

After *de novo* assembly of sequence data for each genome, the number of contigs >100 bp ranged from 44 to 96. By aligning these contigs to the genome of *P. aeruginosa* LESB58 and performing manual genome finishing, contig numbers were greatly reduced, leaving 1 to 9 unresolved gaps per genome. Genomes with the lowest mean coverage, which ranged from 18 to 50 X (Table 1), had more remaining gaps, most of which could be resolved by PCR amplification and Sanger sequencing (results not shown). However, two regions were consistently difficult to assemble with 454 sequencing data alone. Details on these regions and the primers used for gap closure can be found in Table S1. A summary of genome sequencing and assembly as well as accession numbers are shown in Table 1.

**Table 1:** Assembled genomes for 7 *P. aeruginosa* isolates of the Liverpool epidemic strain.

Isolate	Sequencing Technology^1^	Mean coverage	Number of contigs^2^	Assembledlength (bp)	Accession
LES400	454 GS-FLX Titanium	19	4	6590821	CP006982
LES431	454 GS-FLX Titanium	50	1	6550070	CP006937
LESB65	454 GS-FLX Titanium	30	3	6526805	CP006983
LESlike1	454 GS-FLX Plus	18	5	6508670	CP006984
LESlike4	454 GS-FLX Plus	30	3	6523853	CP006985
LESlike5	454 GS-FLX Plus	26	5	6542950	CP006980
LESlike7	454 GS-FLX Plus	23	4	6467614	CP006981

1 Shotgun libraries of the first 3 strains (UK) were sequenced individually on a quarter (LES400, LESB65) or a half (LES431) 454 plate. Shotgun libraries of the last 4 strains (Ontario, Canada) were barcoded and sequenced on a full 454 plate.

2 *De novo* assembly was performed with GS De Novo Assembler (Roche); genome finishing was done with Consed [20].

### Genome Architecture

**Figure 1:**
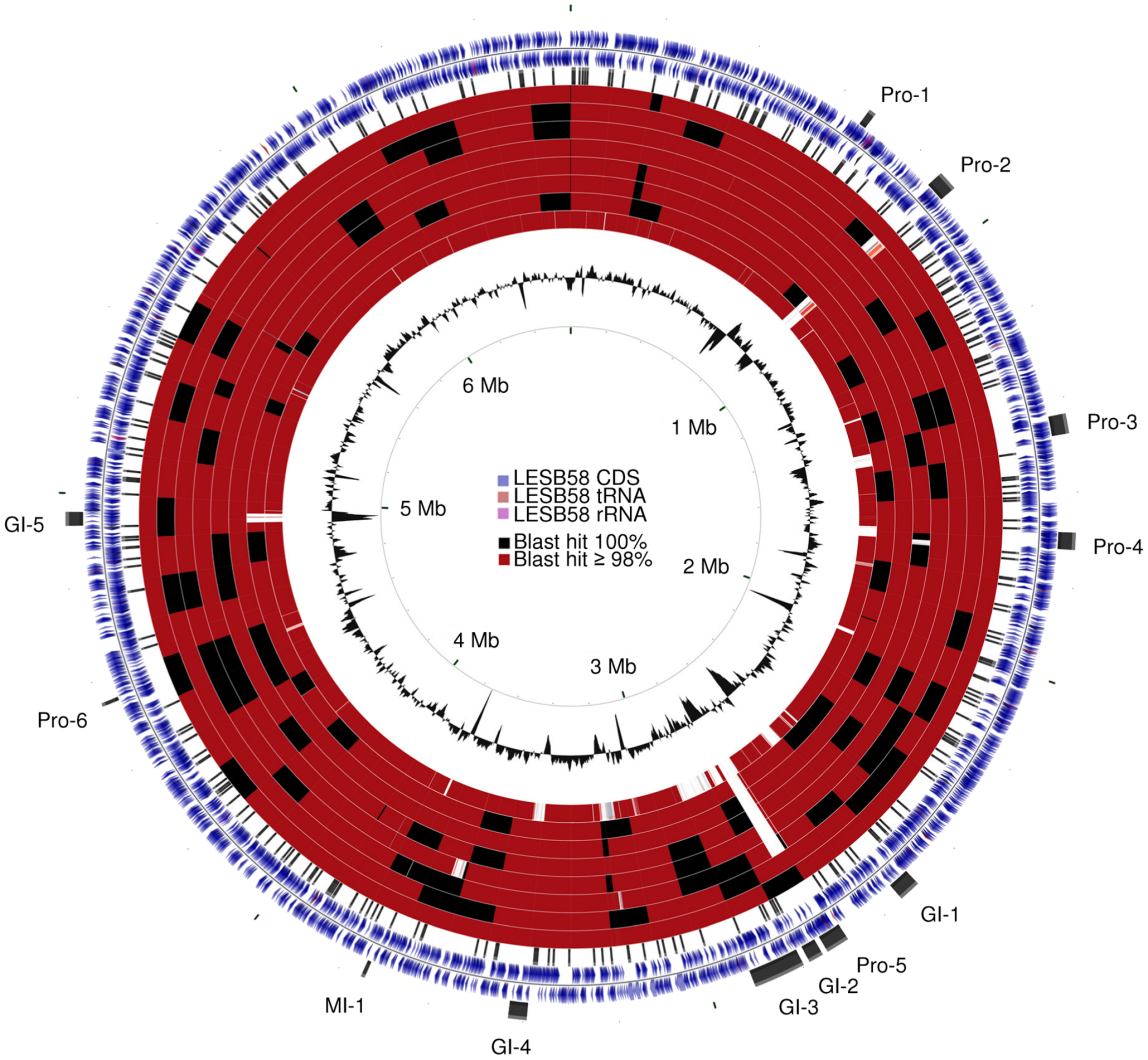
Circular genome map of *P. aeruginosa* LESB58 compared to 7 LES isolates and strain PAO1. Map and underlying analysis were performed with the CGView Comparison Tool [23]. Rings from the outside inward: LESB58 plus-strand, LESB58 minus-strand, 308 unique non-synonymous polymorphisms among LES isolates, LES400, LES431, LESB65, LESlike1, LESlike4, LESlike5, LESlike7, PAO1 and GC content plot. LES prophages (Pro), genomic islands (GI) and LES specific mini island (MI) are indicated on the outside of the map [10]. Orange colored BLAST hits: 90–98% identity, blue colored BLAST hits: 0–90% identity.

Figure 1 contains a circular map of the 7 newly sequenced LES clinical isolates compared to the reference genome of isolate LESB58, and shows that prophages 2 (39 kb) and 5 (48 kb) as well as genomic island 5 (28 kb) are absent from some isolates. More specifically, prophage 2 was absent in LES431 and LESlike7; prophage 5 was absent in LESB65 and all LESlike isolates; and genomic island 5 was absent only in LESlike7. LES400 is the only isolate with no large gap compared to LESB58. Using the Artemis comparison tool, we found that all newly sequenced isolates lacked two copies of pyoverdine biosynthesis genes *fpvA* and *pvdE* compared to LESB58, which carries three copies of these genes (PLES_28981 to PLES_29051). This was later confirmed by PCR assay. Synteny was otherwise conserved among all isolates of this study.

During the course of our analysis, we noted the presence of a previously unreported 7.4 kb LES-specific mini-island that consists of five hypothetical genes from PLES_33841 to PLES_33881, which is not found in any other *P. aeruginosa* genome sequenced to date. Indeed, no orthologs for these LESB58 genes are currently recorded in the genome data of any pseudomonad included in the Pseudomonas Genome Database [33]. As with other genomic islands, the GC content of this island is significantly lower than the average for the whole chromosome (47% versus 66.3%). BLASTn analysis of the entire island did not reveal any significant homology against any bacteria; there may be weak homology at the level of individual proteins, but the genomic context of the island is specific to LES isolates. The only region of homology to any other organism at the nucleotide level is located in the intergenic region between PLES_33831 and PLES_33841, which shares 90% identity with the phage integrase family gene PputGB1_3034 from *Pseudomonas putida*. This observation suggests that the annotation of this island needs to be reviewed and that it is possible that this novel island is the result of defective phage integration or mobilization.

### Core and Accessory Genomes

Panseq [24] was used to isolate the core genome. Genomes of strain PAO1 and LES isolates, including LESB58, were fragmented into 500 bp segments, which had to be present in all 9 genomes in order to be part of the core genome. Any segment that was absent from one or more genomes was added to the accessory genome. Analyses of the core versus the accessory genomes among isolates of the LES clearly demonstrated that the core genome is highly similar among isolates. With a total of 1226 core genome SNPs identified with Panseq (less conservative identification than with CLC Genomics Workbench), clustering and phylogenetic analysis resulted in very short internodes and weakly supported topology (results not shown), except for the group formed by LES400, LES431 and LESB58. Consequently, phylogeographic structure in the data was excluded, similarly to the study by Dettman et al. [34], where LESB58 clustered with and was indistinguishable from Canadian isolates of the LES in a phylogenetic analysis of epidemic and non-epidemic strains of *P. aeruginosa*.

**Figure 2:**
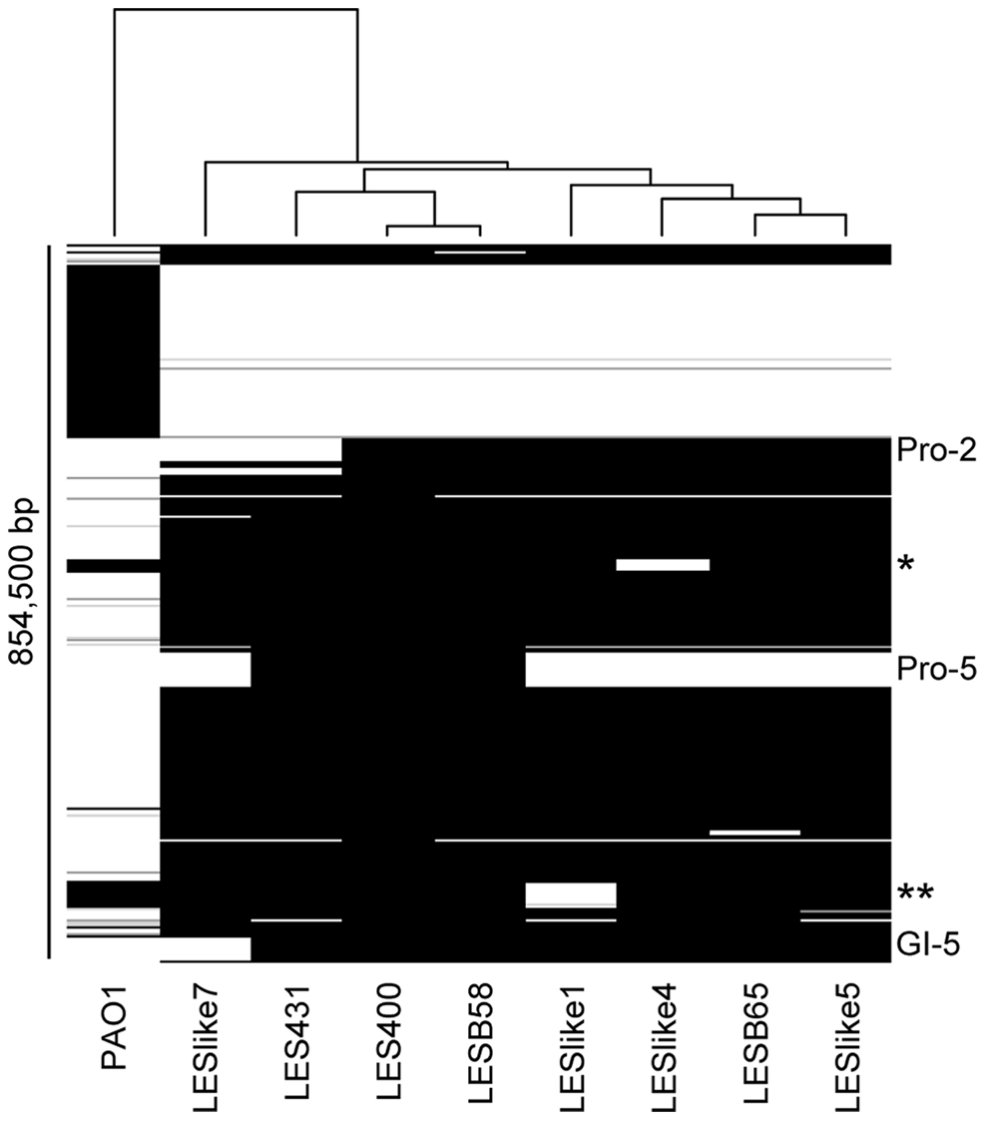
*P. aeruginosa* LES accessory genome dendrogram and heatmap. Strain PAO1 was included as an outgroup. Using Panseq [24], genomes were fragmented in 500 bp segments (default parameters), which were considered as accessory genome if absent from at least 1 of the 9 genomes analysed. Shades of grey represent percent identity, with white = 0% and black = 100%. The dendrogram is based on a hierarchical cluster analysis (Euclidean distance, method = average, r-project version 2.15.1). * Position 1,720,917 in LESB58 (15 kb gap), adjacent to LES prophage 4; ** Position 3,607,181 in LESB58 (32 kb gap).

Accessory element composition on the other hand, was much more diverse. Figure 2 shows that the accessory genomes of LES400 and LES431 are most similar to that of LESB58, while the accessory genome of LESlike7 is clearly distinct from all others. No correlation was established between accessory genome composition and geographic origin. The appearance of this dendrogram is most strongly influenced by prophage and genomic island presence/absence. Two other relatively large regions have an impact on this result (visible on Figure 1): a 15 kb gap in LESlike4 starting at PLES_15971, which contains hypothetical and putative proteins as well as a 5-methylaminomethyl-2-thiouridine methyltransferase (PLES_16041), and a 32 kb gap in LESlike1 starting at PLES_32601, which contains ABC transporter permeases (PLES_32611 and _32621), DNA-binding transcriptional regulator CynR (PLES_32671), carbonate dehydratase (PLES_32681), cyanate hydratase (PLES_32691), RNA polymerase sigma factor (PLES_32711) and serine/threonine transporter SstT (PLES_32801).

### Core Genome Polymorphism

**Figure 3:**
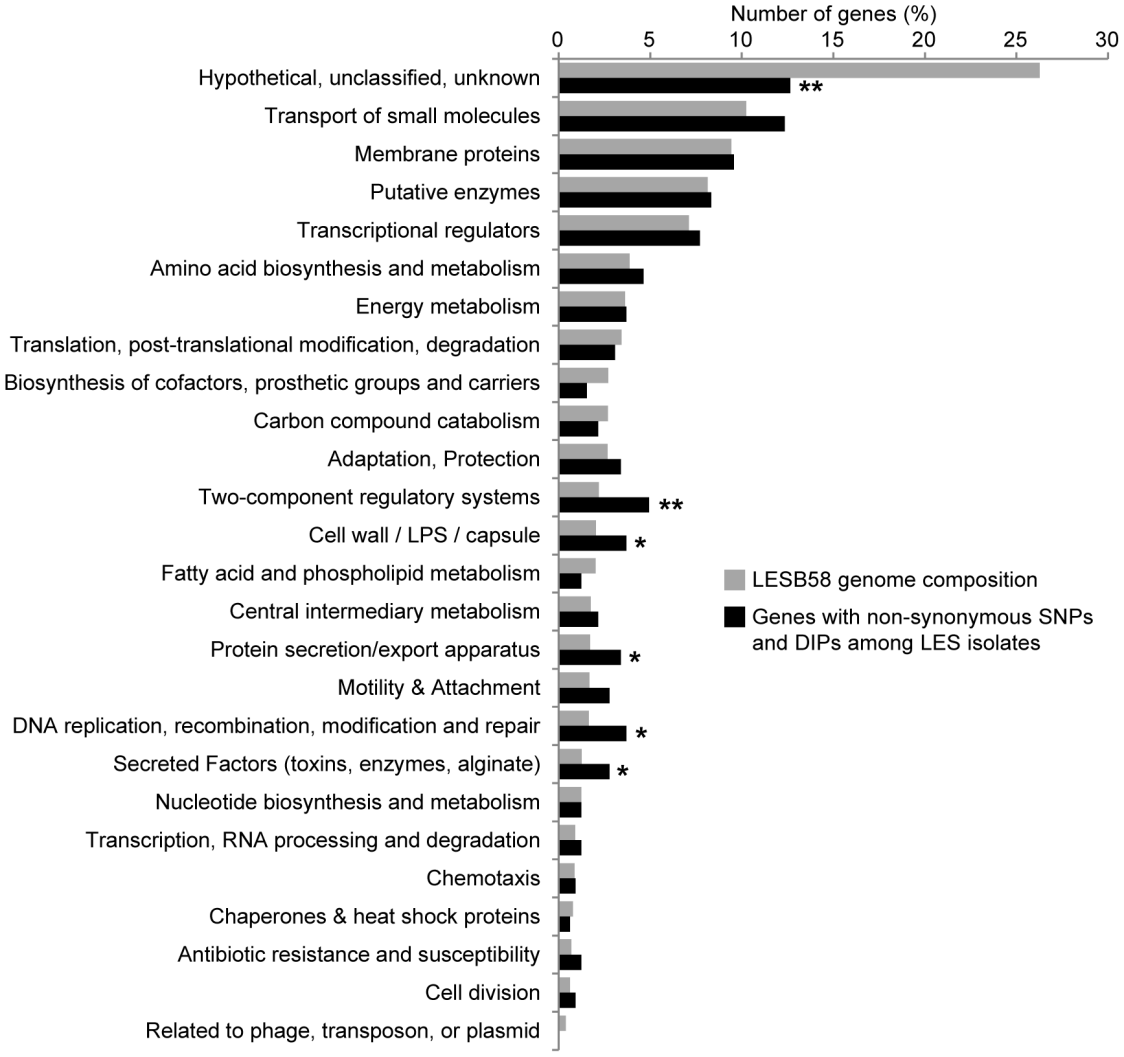
Pseudomonas Community Annotation Project (PseudoCAP) annotation of polymorphic genes among *P. aeruginosa* LES isolates. P-values are from Fisher’s exact tests comparing genes with non-synonymous polymorphisms with LESB58 whole genome composition (* p<0.05, ** p<0.01 & q <0.05).

SNPs and DIPs between each isolate and LESB58 as well as between conserved LES and LES-like sites and PAO1 are summarized in Table 2. The complete and detailed list of polymorphisms among LES isolates is available in Table S2. A total of 308 unique non-synonymous polymorphisms were identified among LES isolates (shown on the circular map, Figure 1), while there were 6391 non-synonymous polymorphisms between conserved LES sites (including LES and LES-like) and PAO1. Non-synonymous polymorphisms were classified according to the Pseudomonas Community Annotation Project (PseudoCAP, [33]). Among LES isolates, polymorphic genes associated with two-component regulatory systems, cell wall, protein secretion, DNA replication and repair, and secreted factors were significantly over-represented compared to LESB58 whole genome composition (Figure 3). Only two-component regulatory systems remained significantly over-represented after correction for multiple testing.

**Table 2:** Summary of SNPs and DIPs among *P. aeruginosa* LES isolates and between LES and laboratory strain PAO1.

Reference	Isolate	SNPs^1^			DIPs^1^		Total
		Non-synonymous	Synonymous	Non-coding	Non-synonymous	Non-coding	
LESB58	LES400	47	16	9	11	3	86
	LES431	37	13	9	5	2	66
	LESB65	70	33	20	6	8	137
	LESlike1	56	21	18	5	9	109
	LESlike4	72	25	25	5	6	133
	LESlike5	84	32	30	2	8	156
	LESlike7	60	26	16	7	7	116
PAO1	LES	5996	19026	3909	974	1118	31023
	LESlike	6028	19174	3914	795	892	30803

1 Polymorphisms were identified with CLC Genomics Workbench (CLCbio) with the following criteria: default parameters for base quality, minimum coverage = 6, minimum frequency  = 0.8 for each new genome against reference LESB58 and 0.95 for all LES or LESlike against reference PAO1.

In order to investigate polymorphism in a way that would be more targeted toward one of the goals of this study, i.e. to associate comparative genomics with virulence characteristics, we specifically extracted non-synonymous polymorphisms that were in genes annotated as virulence factors [35] or implicated in biofilm formation and antibiotic resistance (Table 3). LES isolates had 1 to 4 polymorphisms associated with biofilm formation and 5 to 7 polymorphisms associated with antibiotic resistance, while secretion system (types III and VI) and adherence were the most variable virulence factors. Another central aspect of this study was to identify differences between isolates of the LES from the two continents. Polymorphisms that where conserved among all isolates investigated or specific two one country of origin are summarized in Table 4. Only five SNPs were specific to Canadian isolates, while 15 were specific to UK isolates. However, the most common category was conserved polymorphisms among all isolates compared to LESB58. Most polymorphisms in Table 4 were predicted to have low to moderate functional impact.

**Table 3:** Non-synonymous SNPs and DIPs among *P. aeruginosa* LES isolates that are in genes associated with virulence factors, biofilm formation and antibiotic resistance.

		LESB58 vs.						
		LES400	LES431	LESB65	LESL1^2^	LESL4	LESL5	LESL7
**Adherence** 1		**2**	**2**	**1**		**2**		**1**
	Flagella	1	1	1				
	Type IV pili biosynthesis	1						
	Type IV pili twitching motility related proteins		1			2		1
**Antimicrobial activity** 1					**1**		**1**	
	Phenazines biosynthesis				1		1	
**Antiphagocytosis** 1		**1**					**1**	**2**
	Alginate biosynthesis	1						1
	Alginate regulation						1	1
**Iron uptake** 1				**1**			**3**	
	Pyochelin						1	
	Pyoverdine			1			2	
**Quorum sensing** 1 **systems**		**1**				**1**	**1**	
	N-(3-oxo-dodecanoyl)-L-homoserine lactoneQS system	1				1	1	
**Secretion system** 1		**2**	**1**	**1**	**2**	**1**	**2**	**2**
	Hcp secretion island-1 encoded type VI secretionsystem (H-T6SS)	1					1	1
	P. aeruginosa TTSS^3^	1	1	1	1	1		1
	P. aeruginosa TTSS^3^ translocated effectors				1		1	
**Biofilm**		**2**	**1**	**2**	**3**	**2**	**1**	**4**
**Antibiotic resistance**		**5**	**5**	**5**	**5**	**5**	**7**	**6**

1 According to the Virulence factor database [35], virulence factor associated genes are classified into categories and subcategories.

2 LESL: LESlike.

3 TTSS: type III secretion system.

**Table 4:** Conserved polymorphisms among *P. aeruginosa* LES isolates from the UK and/or Canada.

Category	Reference Position	Putativeimpact^1^	VariationType	Coding Region Change^2^	Amino Acid Change^3^
Specific to UK isolates	4656647	High	SNP	*fleR*:c.316G>T	p.Glu106*
	5095967	High	DIP	PLES_46401:c.9621delC	p.Pro3207fs
	5171	Moderate	SNP	*gyrB*:c.897C>G	p.Ile299Met
	1500912	Moderate	SNP	PLES_13941:c.832G>C	p.Ala278Pro
	1797644	Moderate	SNP	*nosR*:c.550G>A	p.Asp184Asn
	2087274	Moderate	SNP	PLES_19341:c.565G>A	p.Ala189Thr
	3029715	Moderate	SNP	PLES_28161:c.508G>A	p.Gly170Ser
	4875534	Moderate	SNP	PLES_44361:c.311G>C	p.Gly104Ala
	4899936	Moderate	SNP	PLES_44581:c.420C>A	p.Ser140Arg
	5390472	Moderate	SNP	PLES_48951:c.641G>A	p.Gly214Asp
	5902830	Moderate	SNP	PLES_53471:c.1406T>C	p.Val469Ala
	668253	Low	SNP	PLES_06141:c.30G>T	na
	2015427	Low	SNP	na	na
	4807048	Low	SNP	na	na
	5019644	Low	SNP	na	na
Specific to Canadian isolates	1213228	Moderate	SNP	*pcs*:c.80C>T	p.Thr27Ile
	2261764	Moderate	SNP	*rne*:c.508A>G	p.Ser170Gly
	5222650	Moderate	SNP	PLES_47391:c.95G>A	p.Gly32Asp
	4095795	Low	SNP	na	na
	4993560	Low	SNP	*prpB*:c.702A>G	na
Conserved among all isolates compared to LESB58	123796	Moderate	SNP	PLES_01041:c.125T>C	p.Leu42Pro
	424909	Moderate	SNP	*metX*:c.602C>T	p.Thr201Ile
	594120	Moderate	SNP	PLES_05421:c.304C>A	p.Pro102Thr
	1211690	Moderate	SNP	PLES_11151:c.26T>C	p.Val9Ala
	1676171	Moderate	SNP	PLES_15441:c.1004T>C	p.Val335Ala
	2076950	Moderate	SNP	PLES_19231:c.307A>G	p.Thr103Ala
	3201324	Moderate	SNP	PLES_29121:c.494T>C	p.Val165Ala
	3339113	Moderate	SNP	PLES_30291:c.259T>G	p.Cys87Gly
	3478510	Moderate	SNP	PLES_31551:c.294G>C	p.Leu98Phe
	5039965	Moderate	SNP	PLES_45841:c.346A>G	p.Thr116Ala
	5369024	Moderate	SNP	PLES_48781:c.752A>T	p.Glu251Val
	5370379	Moderate	SNP	PLES_48791:c.436A>C	p.Ile146Leu
	5398854	Moderate	SNP	*ampD*:c.224T>C	p.Phe75Ser
	5763379	Moderate	SNP	PLES_52231:c.898T>G	p.Cys300Gly
	5781270	Moderate	SNP	*fis*:c.112T>G	p.Tyr38Asp
	5816665	Moderate	SNP	*desB*:c.748G>A	p.Ala250Thr
	6076482	Moderate	SNP	PLES_54891:c.1159C>G	p.Pro387Ala
	6483542	Moderate	SNP	PLES_58511:c.602A>G	p.His201Arg
	3376895	Low	SNP	*pslK*:c.855T>C	na
	3997423	Low	SNP	na	na
	5625198	Low	SNP	*phuR*:c.2160T>C	na
	6473649	Low	SNP	*wbpY*:c.507T>C	na

1 “High” for nonsense (*) or frame-shifting (fs) mutations, “Moderate” for amino acid substitutions, “Low” for silent coding and non-coding mutations.

2 c.: position in the gene sequence.

3 p.: position in the protein sequence.

As there was so little genetic variability among LES isolates, we hypothesized that regulatory variation is likely to play an important role in LES phenotypic diversity. Table 5 summarizes all 52 unique non-synonymous polymorphisms that were in genes with PseudoCAP terms “two-component regulatory systems” and “transcriptional regulators”. Fifteen of these cause a stop codon or a frame shift in the protein sequence; hence they are very likely to have biological impact.

**Table 5:** Non-synonymous SNPs and DIPs among *P. aeruginosa* LES isolates found in regulatory genes.

Gene^1^	Function^2^	LES400	LES431	LESB65	LESL1^3^	LESL4	LESL5	LESL7
*ampR*	Transcriptional regulator, LysR substrate binding domain	1	1					
*chpA*	Still frameshift putative component of chemotactic signal transductionsystem (Adherence: Type IV pili twitching motility related proteins)					1*		1*
*cysB*	Transcriptional regulator, LysR substrate binding domain			1				
*exsA*	Bacterial regulatory helix-turn-helix proteins, AraC family(Secretion system: TTSS)		1	1	1	1		
*fis*	Bacterial regulatory protein, Fis family	1	1	1	1	1	1	1
*fleR*	Response regulator (Adherence: Flagella)	1*	1*	1*				
*hutC*	Bacterial regulatory proteins, gntR family			1		1		
*kdpD*	Osmosensitive K+ channel histidine kinase			1				
*ladS*	Signal transduction histidine kinase					1		
*lasR*	LuxR family transcriptional regulator (Quorum sensing systems:N-(3-oxo-dodecanoyl)-L-homoserine lactone QS system)	1*				1	1*	
*metR*	Transcriptional regulator, region: LysR			1		1		
*mucA*	Negative regulator of sigma E activity(Antiphagocytosis: Alginate regulation)							1*
PLES_02631	Predicted signal transduction protein						1	
PLES_03151	Uncharacterized protein conserved in bacteria			1			1	1*
PLES_04751	Transcriptional regulator	1*						
PLES_08711	Osmolarity response regulator	1*						1
PLES_09021	Putative transcriptional regulator					1		
PLES_09811	Transcriptional regulator, LysR substrate binding domain	1						
PLES_10561	ATP-dependent transcriptional regulator, region MalT	1						
PLES_17961	Phosphate regulon sensor kinase PhoR						1	
PLES_18611	Putative two-component sensor				1		1	
PLES_19821	Putative two-component sensor					1		
PLES_21321	Transcriptional regulator, region: AcrR						1	
PLES_24391	Anaerobic nitric oxide reductase transcription regulator		1					
PLES_26001	Bacterial regulatory helix-turn-helix proteins, AraC family				1			
PLES_28161	Putative two-component sensor	1	1	1				
PLES_31481	Histidine Kinase A					1		
PLES_34121	RNA polymerase sigma factor; Sigma-70 region 2	1*						
PLES_37161	Histidine kinase-like ATPase					1		
PLES_38451	Histidine kinase-like ATPase							1
PLES_38731	Aminoethylphosphonate catabolism associated LysR familytranscriptional regulator						1	
PLES_39221	Transcriptional regulators of sugar metabolism				1			
PLES_48791	DNA-binding transcriptional repressor PuuR	1	1	1	1	1	1	1
PLES_58801	Phosphate regulon sensor kinase PhoR				1			
*pmrA*	DNA-binding transcriptional regulator							1*
*pmrB*	Two-component regulator system signal sensor kinase	1*				1		
*rpoN*	RNA polymerase factor sigma-54					1*	1	
*vfr*	cAMP-regulatory protein; DNA binding domain ofprokaryotic regulatory proteins							2*

*indicates that the polymorphism introduces a stop codon or a frame shift in the protein sequence.

1 Genes selected have a PseudoCAP annotation that includes terms “Transcriptional regulators” and/or “Two-component regulatory systems” (pseudomonas.com).

2 Virulence factor database annotation is indicated between parentheses when applicable.

3 LESL: LESlike.

### Strain Phenotyping

As expected, most phenotypes investigated showed extensive variability among isolates. Exceptions include hemolytic activity and H_2_O_2_ susceptibility, which were similar in all isolates (results not shown). In addition, all isolates were non-mucoid. Upon plating in preparation for phenotyping experiments, isolate LESlike1 had two distinct colony morphologies, one likely a small colony variant of the other (referred to as LESlike1s hereafter). LESlike1s was not observed during sample preparation for genome sequencing, and no more ambiguous SNPs (frequency <80%) were identified in the resulting sequence data compared to the other isolates. Hence the sequence data most likely corresponds to LESlike1. LESlike1 and LESlike1s were isolated and treated separately for phenotypic experiments.

**Figure 4:**
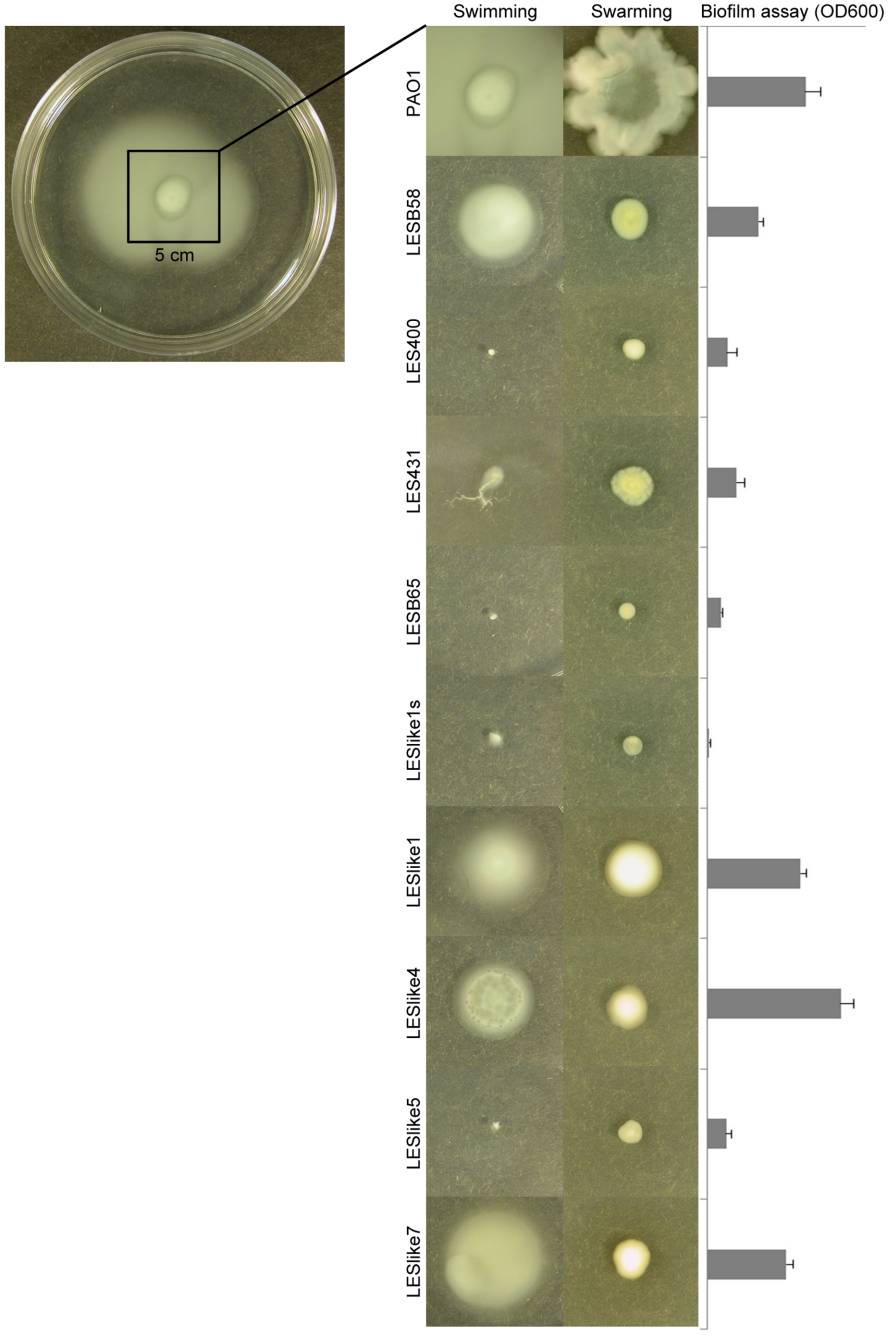
Swimming, swarming and biofilm formation. Swimming and swarming: the isolate was considered non-motile when bacterial growth could be observed only at the site of inoculation. Biofilm: mean and standard deviation from 8 wells in a single 96-well microplate assay. Results shown are from 1 of 3 replicate experiments.

Phage plaques were fortuitously observed on two occasions during phenotyping: in the H_2_O_2_ susceptibility test for LESlike1s and in the swimming test for LESlike4 (visible in Figure 4). Phages were isolated from plaques for future studies.

### Motility and Biofilm Formation

While all LES isolates were negative for twitching motility, swimming and swarming were observed in LESB58, LESlike1, 4 and 7 (Figure 4). However, motility levels were much weaker than in reference strain PAO1. As depicted in Figure 4, motility and biofilm production appeared to be related among LES isolates.

### Proteolytic and Elastolytic Activity

**Figure 5:**
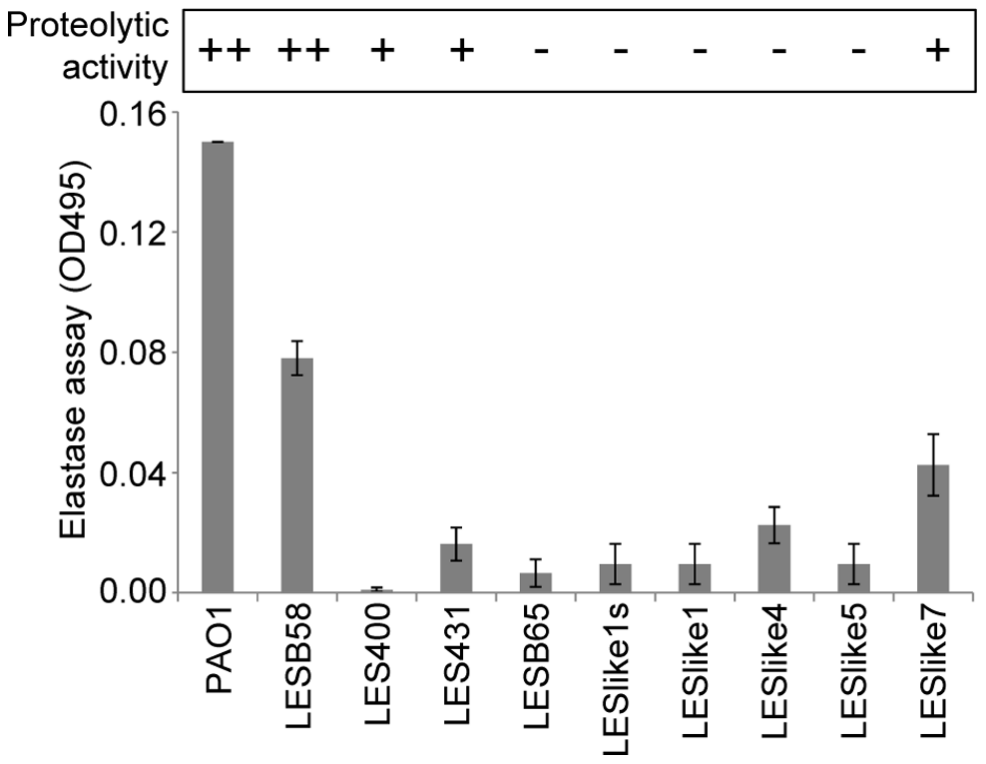
Proteolitic and elastolytic activity in culture supernatants. Proteolytic activity was measured using BHI agar plates containing 1% skim milk; - no activity,+small halo of proteolysis,++halo diameter multiple times that of the culture supernatant sample. Results were reproducible. Elastolytic activity was determined with the Elastin-Congo Red Assay after 3 hours. Measurement values varied greatly between experiments, hence data was normalized so that OD495 = 0.15 for PAO1 in all experiments, error bars: standard deviation from 3 independent experiments.

Figure 5 shows results for both observed proteolysis on BHI agar +1% (wt/vol ) skim milk measuring exoprotease secretion and an elastin-congo red assay measuring LasB elastase activity in culture supernatants [36]. There was general concordance between these two phenotypes. LESB58 clearly showed more proteolytic and elastolytic activity compared to all other LES isolates.

### Pyocyanin and Pyoverdine Secretion

**Figure 6:**
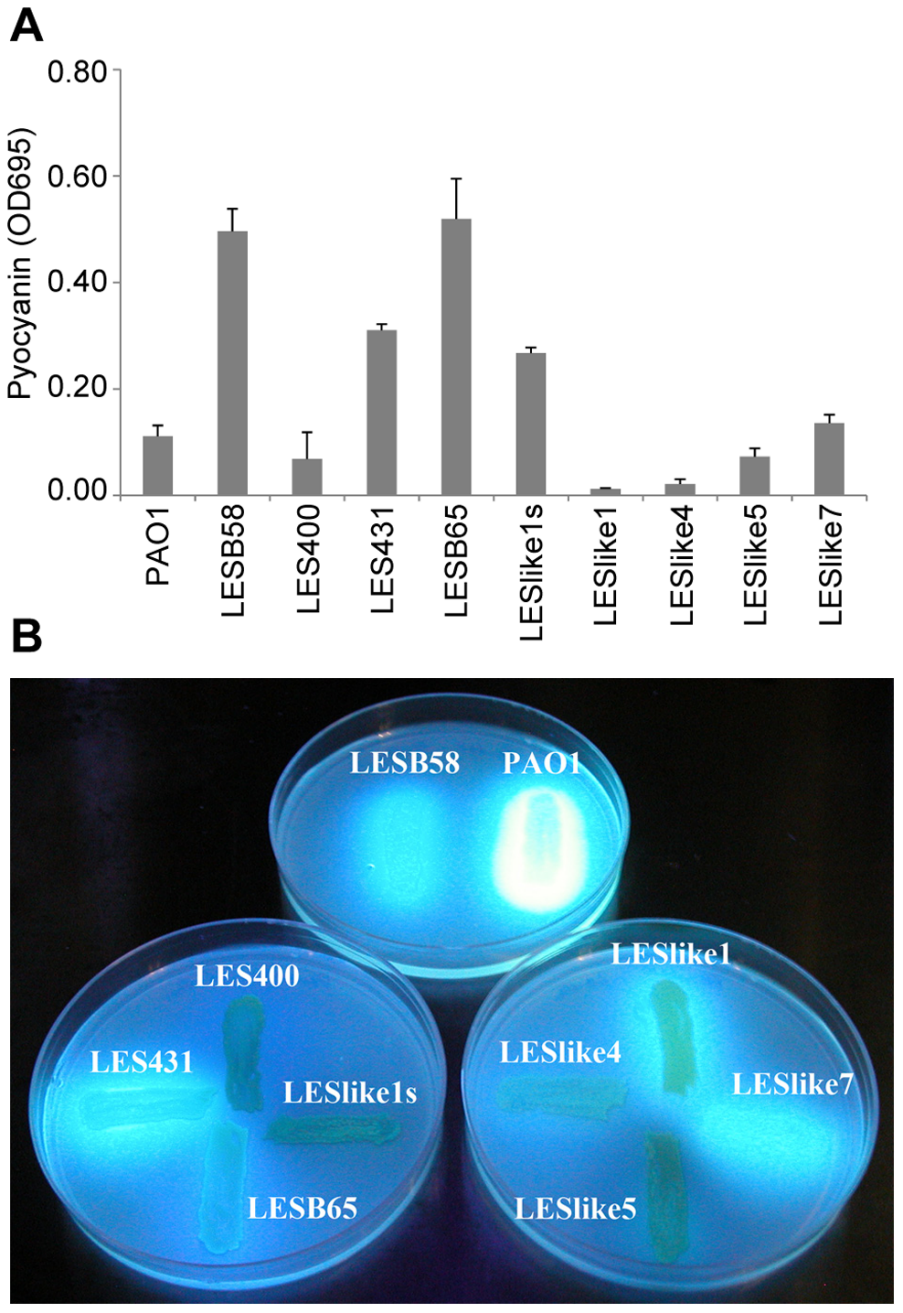
Pyocyanin and pyoverdine production. A. Measurements of pyocyanin from overnight culture supernatants, error bars: standard deviation from 3 independent experiments. B. Pyoverdine production on Pseudomonas agar F (Difco) visualized under long wavelength UV light, results shown are from 1 of 3 replicates.

Figure 6 presents a comparison of pyocyanin and pyoverdine production. Pyocyanin results have been normalized for bacterial culture density. Raw results showed the same pattern, except that LES431 and LESB65 both had OD695>0.6. LESB58, LES431, LESB65 and LESlike1s over-produced pyocyanin compared to strain PAO1. Isolates LESB58, LES431, LESlike1 and LESlike7 produced the most pyoverdine while LES400, LESlike1s and LESlike5 did not produce any. LESB65 and LESlike4 had intermediate production.

### Phenotypic Microarray

Significant results of the phenotypic microarray experiment comparing LES431 with LESB58 are presented in Table S3. Briefly, exploitation of 22 dipeptide and nitrogen compounds was significantly lower in LES431 compared to LESB58.

### Predation Assay in the Amoeba

**Figure 7:**
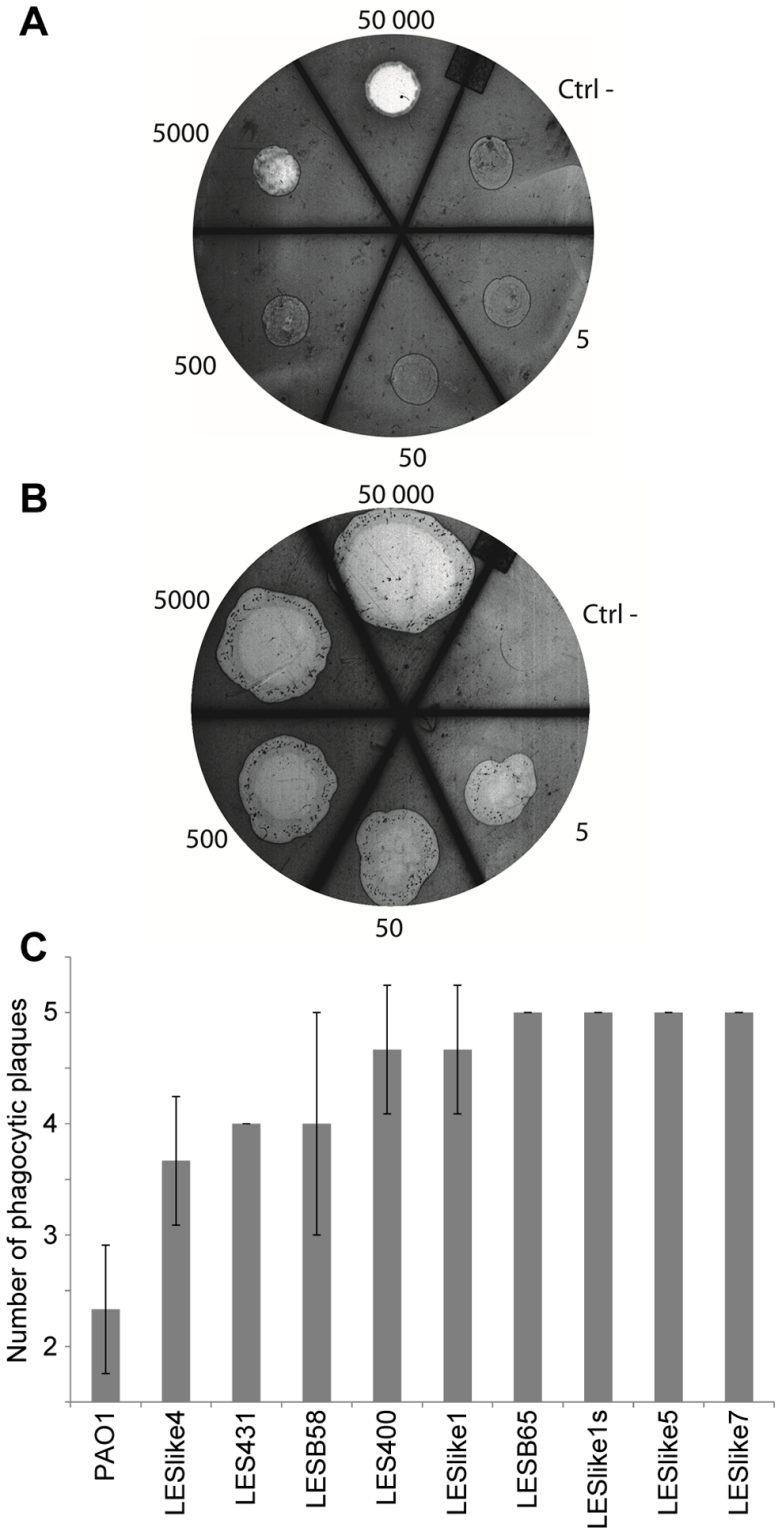
Amoeba virulence assay. A. Example of *P. aeruginosa* strain PAO1 showing 2 phagocytic plaques. Numbers represent the total number of amoebae deposited on the bacterial lawn. B. Example of *P. aeruginosa* isolate LES400 showing 5 phagocytic plaques. C. Mean and standard deviation from 3 independent experiments. PAO1 is considered as an amoeba-resisting bacterium (average number of phagocytic plaques <3).

According to a predation assay using amoebae, only strain PAO1 could be considered as resistant to amoeba grazing (number of phagocytic plaques <3). In fact, the virulent wild-type strain PAO1 was shown to inhibit growth of *D. discoideum* [30]. In contrast, all LES isolates were considered as avirulent in this host model (number of phagocytic plaques >3). Still, we observed significant variability among them (Figure 7, Kruskal-Wallis rank sum test on LES data only p  = 0.025), as LESB58, LES431 and LESlike4 showed intermediate levels of resistance to amoeba grazing compared to resistant strain PAO1 and unequivocally susceptible isolates (number of phagocytic plaques  = 5 in all replicates). Pairwise comparisons using Wilcoxon rank sum test confirmed significance of this difference (p<0.05) for LES431 and LESlike4.

## Discussion

### Genome Sequencing and Assembly

According to our experience, mean genome coverage of 30 or greater was ideal for ease of automated and manual assembly. Still, we encountered multiple so-called intractable regions, which have been demonstrated to form GC-rich secondary structures that are refractory to amplification and sequencing [37]. This issue was discussed at length by Jeukens *et al.* [18]. In addition, region B (Table S1) contained repeated DNA segments, which greatly interfered with specific primer design for PCR amplification and Sanger sequencing.

### Accessory Genome Diversity

The first most striking comparative genomics result presented in this study is the variability in prophage and genomic island complement among isolates. It was previously shown that LESB58 with insertion mutations in genomic island 5, prophages 2, 3 or 5 had 10 to 1000-fold decreased competitiveness in a rat model of chronic lung infection when compared to wild-type LESB58 [10]. These results demonstrated the importance of these accessory regions for *in vivo* maintenance of the LES during chronic infection. Prophages and genomic islands present in genomes of the 3 newly sequenced LES isolates corroborate previous PCR-based identification [13]. However, genomic island 4 was present in all LES isolates, which contradicts previous results and demonstrates the inadequacy of the proposed PCR assay to detect this island [13]. In a study of genomic fluctuation within *P. aeruginosa* populations in the CF lung during very short episodes of exacerbations and antibiotic therapy, typical prophage and genomic island complements tended to reflect the results of this study [38]. Here, prophage 5 was absent in 5 isolates, prophage 2 was absent in 2 isolates and genomic island 5 was absent in a single isolate. Fothergill *et al.* [38] found that, while prophage 5 was absent from all LES isolates investigated, prophages 2–4 were detected in all isolates prior to treatment, but loss of these prophages was observed in isolates from one patient following antibiotic therapy. LES prophages 2 and 3 have been detected as active phages in sputa of infected patients [39]. As for genomic island 5, it’s prevalence was either increasing or stable at a high level among LES isolates throughout treatment, which led the authors to suggest positive selection for this accessory region [38]. The role played by bacteriophages and genomic islands in CF lung infections clearly needs further investigation, but current evidence suggests that accessory genome regions, even those that have been shown to increase competitiveness, are not necessarily essential as lung infection evolves [11], [13], [38].

### Core Genome Diversity

The first goal of this study was to investigate genomic diversity among isolates of the LES. Strain PAO1 was included as a non-epidemic reference genome. A detailed investigation of the evolutionary genomics of epidemic versus non-epidemic strains was done by Dettman et al. [34]. Of the 308 unique non-synonymous polymorphisms among LES isolates identified in this study, 284 were in regions of the core genome shared with strain PAO1 and all were in regions shared by all LES isolates. LES400 and LES431, which are the oldest isolates sequenced in this study, were more closely related to LESB58 compared to the other isolates. LES431 is distinct from other isolates of this study in that it was implicated in unusual infection of the non CF parent of a CF patient [15]. It was isolated shortly after the onset of infection and can therefore be thought of as a relative of other LES isolates that is better adapted to initiate an infection, even in a non-CF environment. LES431 also had the fewest polymorphic sites compared to LESB58, the earliest LES isolate. Lower genomic variation among clonal isolates with less diverse spatiotemporal origin was observed in a previous study of *P. aeruginosa* isolates derived from common and divergent sources [40]. As we have identified no evidence of phylogeographic structure in this study, it is possible that the relatedness among LESB58, LES400 and LES431 is due to proximity in time rather than in space.

Intraclonal diversity and evolution in the CF lung were investigated in multiple *P. aeruginosa* strains, and variation typically translates to a few dozen SNPs and DIPs in the core genome, sometimes accompanied by a large deletion or inversion. Yang *et al.* [41] have sequenced the genomes of 12 strains of the transmissible DK2 lineage isolated between 1973 and 2008 and found a total of 368 SNPs as well as 180 SNPs between the most distantly related clones, which is in the same order of magnitude as the data presented here. Similarly, among two members of the PA14 clonal complex, 231 SNPs were identified and non-synonymous SNPs were mainly found in genes involved in transcriptional regulation, membrane and extracellular constituents, transport, and secretion [8]. Among 34 genes that have been identified as mutational targets during airway infection for 29 CF patients [9], 21 had non-synonymous polymorphisms in this study. Of these, 14 were identified between conserved LES sites and strain PAO1, including *mexS*, *mexT*, *mexZ*, *accC*, *wspF*, *nalD*, *pilB*, *rhlI*, *toxR* and *ampD*. Only 7 were identified among LES isolates, namely in *lasR*, *vfr*, *exsA*, *rpoN*, *cyaB*, and *ampD* (see Table S2 for details). Except for *ampD*, only 1 to 4 of the 7 newly sequenced isolates differed from LESB58. In another study focussing on intraclonal variation in the clone C lineage, only 2 of the 12 most frequent mutational targets were polymorphic: *mexA* and *exsA* [42]. These results reflect core genome heterogeneity among *P. aeruginosa* CF isolates, which can even be observed among isolates collected from a single patient, at a single time point [9], [12].

### Regulatory Variation among Isolates of the LES

It is thought that *P. aeruginosa* owes its metabolic versatility in part to its large genome as well as its >543 transcriptional regulators and two-component regulatory systems [8], [43]. The latter was the most significantly enriched functional category in genes with non-synonymous polymorphisms among isolates of the LES. Two-component regulatory systems allow bacteria to sense and respond to environmental cues [44]. *P. aeruginosa* possesses a large array of such systems, which are key to modulate virulence and antibiotic resistance processes (reviewed by [45]).

Approximately one out of six non-synonymous polymorphisms among LES isolates of this study was in a regulatory gene. Only two were shared by all newly sequenced isolates (in genes encoding Fis and PuuR), but are unlikely to alter protein function. Other most frequently variable proteins included ExsA and FleR. ExsA is a transcriptional activator of type III secretion and differed from LESB58 in four isolates, each with a different amino acid substitution (LES431, LESB65, LESlike1 and LESlike4). The three UK LES isolates shared a nonsense mutation in regulatory gene *fleR*. FleR mutants have no visible flagellum and are non-motile [46], as is the case for these isolates [15]. UK isolates also shared another potentially relevant regulatory polymorphism: all three had a 14 bp deletion in *gltR*, which is essential for glucose transport [47]. This deletion, which did not meet quality requirements during DIP identification, was confirmed by manual inspection of the original genome assemblies in Consed. Fixed mutations in global regulators LasR, RpoN and MucA have been identified in *P. aeruginosa* transmissible lineage DK2 [41]. We also identified mutations in these proteins; none were fixed, but most likely affect protein function. LasR, which is a key regulator of quorum sensing, was mutated in LES400 (previously identified insertion [15]), LESlike4 (amino acid change) and LESlike5 (nonsense mutation). MucA, which is implicated in alginate regulation, had a frame shifting insertion in LESlike7 only and RpoN was mutated in LESlike4 (deletion) and LESlike5 (amino acid change, unlikely to affect protein function). RpoN mutants normally show severe growth defects that can only be partially complemented with glutamine [48], which was not the case for any isolate in this study. The raw sequence data for this gene was manually verified in LESlike4: although coverage and frequency of the mutation were acceptable, sequence assembly quality is fairly low in the region surrounding the deletion.

The majority of these polymorphisms were isolate-specific, hence regulatory variation is likely to be an important component of observed variability among LES isolates and will require further investigation in the near future. A microarray experiment has been performed to compared gene expression levels among strain PAO1, isolate LES400 and isolate LES431. The most important result was that the majority of genes overexpressed in LES431 were genes known to be activated by quorum sensing [15]. LES400 on the other hand, is QS-deficient due to its mutated regulator LasR. RNA sequencing (RNA-Seq) experiments on bacteria harvested directly from the lung in a chronic infection model should provide insight into biologically relevant variation in gene expression among LES isolates.

### LES Phenotypic Diversity and Virulence

As previously observed in clinical isolates of the LES within and among patients, we observed great phenotypic variability among the 7 isolates of this study [12]. This is the first report of motility in the LES, which is usually described as non-motile [15]. However, the impact on the host of the limited swimming and swarming activities observed in this study, if any, is unclear. Moreover, biofilm results for the three newly sequenced Liverpool isolates were inconsistent with previously published results [13]. Replication of this assay by two experimenters in different laboratories clearly showed that discrepancies could not be attributed to variations in experimental conditions alone. Sequenced isolates have undergone very limited manipulation in the lab, but divergence from original isolates cannot be excluded.

QS overproducers are frequent among LES isolates [12], [14] and can be easily recognized by the overproduction of phenazine pyocyanin compared to strain PAO1. LES400 is a known LasR mutant and transcriptomics experimentation has confirmed that the QS regulon is down-regulated [15]. LES431, LESB65 and LESB58 are known to produce high levels of quorum sensing regulated activity [13], [15]. Results presented here are in line with these two observations. Among Canadian isolates, only LESlike1s could be considered as a QS overproducer, while LESlike 5 and LESlike 7 showed measurements similar to those for PAO1. Therefore, the LESlike5 LasR mutation mentioned earlier appears to reduce but not abolish pyocyanin production. A genetic basis for the QS overproducing phenotype, on the other hand, was never identified [15]; we found 3 conserved amino acid changes between the LES and strain PAO1 in genes implicated in quorum sensing (PA0005 and PA3476) and 6 in genes implicated in phenazine biosynthesis (PA0051, PA1899, PA4211 and PA4217), but all are most likely silent. No specific mutation could be associated with the observed patterns of protease, elastase and pyoverdine secretion among LES isolates.

It was previously observed that the frequency of auxotrophic mutants among LES populations tended to increase during pulmonary exacerbation, presumably because amino acid concentration in the lung environment is especially high during exacerbation and prototrophy is metabolically costly [38]. The phenotypic microarray experiment showed significant reduction in the use of certain dipeptide and nitrogen compounds by LES431 compared to LESB58. As mentioned above, LES431 was implicated in a very uncommon acute infection of the non CF parent of a CF patient; hence these results could be in line with the hypothesis of costly prototrophy. Polymorphisms in *nirB* (nitrite reductase), PLES_24391 (anaerobic nitric oxide reductase transcription regulator), and PLES_48781 (aminopeptidase) could be linked to observed differences. Namely, polymorphism in *nirB* could be significant because residue C642 is a ligand for the coordination of the iron-sulphur cofactor of the enzyme.

It can be very difficult to predict virulence of a strain solely based on *in vitro* expression of individual virulence factors. Using an infection model is an effective way of evaluating the aggressiveness of a pathogen toward a specific host. Some of the isolates of this study have previously been included in this type of experiment. In the murine model of acute respiratory infection, there was a virulence hierarchy of LES431>LESB65>LESB58>LES400 [13], and in the fruit fly *Drosophila melanogaster*, LES431 was more virulent than LES400, which was more virulent than strain PAO1 [15]. Results obtained using the amoeba predation model are different, but are in line with the previous demonstration that CF isolates of *P. aeruginosa* are generally less virulent than isolates from other sources in this host model [49], [50]. Interestingly, isolate LES431 was more resistant to amoeba predation compared to most LES isolates. Information on the duration of infection is not known for LESlike4, which was also significantly more resistant than most LES isolates. In a previous study [50], it was shown that *P. aeruginosa* has the tendency to lose its capacity to resist amoeba predation after a long period in the CF lung suggesting that adaptation to a chronic persistent lifestyle in the lung is associated with a certain loss of virulence compared to recently transmitted and/or acute infection isolates, which retain resistance capacities.

Overall, we observed a lack of correlation among phenotypes. This had already been demonstrated in another study of clinical isolates of the LES [12] and in a recent study focussing on the Prairie epidemic strain (PES), where phenotypes displayed high variability, but were generally independent from one another [51]. The same study showed an absence of correlation between genetic and phenotypic clustering of isolates, which is also in line with results presented here.

### Transcontinental Evolution of the LES in CF Chronic Lung Infections

With this study, we were interested in uncovering genomic and phenotypic traces of the transcontinental transmission of the LES. This was challenging, as isolates of this study were very closely related and, for all intents and purposes, phylogenetically undistinguishable. Most conserved polymorphisms were low to moderate impact SNPs shared among all isolates compared to LESB58, hence they probably date back to early evolution of the strain. Likewise, at the phenotypic level, isolates from both origins were equally variable with no evidence for divergence. On the other hand, 29% of regulatory mutations were identified as having potentially high functional impact, and most regulatory mutations were isolate-specific. Therefore, polymorphism in regulatory genes is likely to be an important basis for phenotypic diversity among LES isolates.

Traditionally speaking, it is thought that during the transition from the initial acute infection to a persistent chronic lifestyle in the CF lung, *P. aeruginosa* adapts to its new environment by switching to a mucoid [52], non-motile [53], resistant phenotype [54]. Specific mutations have been associated with the general loss of virulence effectors that mark this transition and presumably evolves to evade the host’s immune defenses [9]. Current knowledge of transmissible *P. aeruginosa* strains, including this study, clearly demonstrates that this view of adaptation to the CF lung is overly simplified. It is now obvious that these strains undergo rapid microevolutionary diversification in the CF lung, leading to a complex array of subtypes from a single clonal lineage, in a single patient [12], [13], [40], [51]. Multiple studies have reported significant phenotypic variability of isolates within patients. For instance, Workentine *et al.* [51] observed almost equal proportions of motile and non-motile isolates, as well as mucoid and non-mucoid isolates in the PES. In the LES, isolates differ in auxotrophy, hypermutability, antibiotic resistance and virulence factor production, which is either lost, maintained or intensified (e.g. pyocyanin) [12]. However most LES isolates are non-mucoid [38].

Heterogeneity of the CF lung promoting adaptive radiation might be responsible for this phenotypic diversity [55]. Furthermore, standing genetic diversity, which does not necessarily contribute to phenotypic diversity (i.e. cryptic variation), might fuel rapid adaptive change in the face of fluctuating conditions in the lung [56]. Thus, understanding population dynamics could be a key element to understand evolution of the LES in the CF lung [12], [38]. Even though *P. aeruginosa* does not seem to require many *de novo* mutations in order to adapt to a new environment [8], identifying the genetic basis for adaptation to the CF lung and transmissibility among patients remains a daunting task. Access to a larger set of complete *P. aeruginosa* genomes should enhance the capacity of comparative genomics analyses to identify relevant genetic features.

## Supporting Information

Table S1 **Primers for amplification and Sanger sequencing of two regions that are consistently difficult to assemble with 454 sequencing alone in** ***P. aeruginosa*** **LES.**
(PDF)

Table S2 **All SNPs and DIPs among** ***P. aeruginosa*** **LES isolates.**
(XLSX)

Table S3 **BIOLOG phenotypic microarray significant results for isolate** ***P. aeruginosa*** **LES431 compared to LESB58.**
(PDF)
