# Supplementary material for: Comparative Genomics of Isolates of a Pseudomonas aeruginosa Epidemic Strain Associated with Chronic Lung Infections of Cystic Fibrosis Patients
Source: PLoS One. 2014 Feb 5;9(2):e87611. doi: 10.1371/journal.pone.0087611 (PMC3914812; doi:10.1371/journal.pone.0087611)
Supplement: Table S1 — Primers for amplification and Sanger sequencing of two regions that are consistently difficult to assemble with 454 sequencing alone in P. aeruginosa LES. (PDF) [file pone.0087611.s001.pdf]

Table S1. Primers for amplification and Sanger sequencing of two regions that are consistently difficult to assemble with 454 sequencing alone in *P. aeruginosa* LES.

Region	LESB58 position	Amplicon size (bp)	Forward primer	Reverse primer	Additional primer(s)	Protocol
A1	3824447	3019	ACTGGTCCCAACTGAAGTCGAT	CAATACCAGCCTTCCCGCTAC	CGATACGCACCTGACTGTTCG<br>AACAACAGCAGTCCCACCAG	Qiagen HotStarTaq
A2	3827429	2798	AGGAATCCACCGTCGTGGTAG	GTAGCCAACTGCCGGATG	GATGACGACGCCGTTGCT<br>CAGTGCGGGCAGCAGTGT	Qiagen HotStarTaq
B	5521541	4060	AAAGGGTTAGAAATTCAGAGGCTTG	AGATGTTCAAGACCACCACCACT	CATAGTTGCCACTGGTCAGCA	Feldan FastPfu

Additional primers are meant for additional sequencing reactions to cover amplicon centers.
