# Supplementary material for: Comparative Genomics of Isolates of a Pseudomonas aeruginosa Epidemic Strain Associated with Chronic Lung Infections of Cystic Fibrosis Patients
Source: PLoS One. 2014 Feb 5;9(2):e87611. doi: 10.1371/journal.pone.0087611 (PMC3914812; doi:10.1371/journal.pone.0087611)
Supplement: Table S3 — BIOLOG phenotypic microarray significant results for isolate P. aeruginosa LES431 compared to LESB58. (PDF) [file pone.0087611.s003.pdf]

Table S3. BIOLOG phenotypic microarray significant results for isolate *P. aeruginosa* LES431 compared to LESB58.

Chemical	Mode of Action	Time	P-value (<1E-03)	LES431 vs. LESBB58
Ala-Lys	Dipeptide	24h	9.9E-04	down
Arg-Glu	Dipeptide	24h	4.2E-05	down
Arg-Glu	Dipeptide	26h	2.5E-04	down
Arg-Glu	Dipeptide	30h	4.6E-04	down
Arg-Ile	Dipeptide	30h	8.9E-04	down
Asp-Ala	Dipeptide	24h	1.0E-04	down
D,L-Lactamide	Dipeptide	24h	5.3E-04	down
His-Leu	Dipeptide	24h	6.9E-04	down
His-Leu	Dipeptide	26h	7.7E-04	down
His-Leu	Dipeptide	30h	5.8E-04	down
Ile-Leu	Dipeptide	30h	2.7E-04	down
Tyr-Val	Dipeptide	26h	2.5E-07	down
a-Amino-N-Valeric Acid	Nitrogen	30h	3.0E-05	down
Acetamide	Nitrogen	24h	8.6E-04	down
Adenosine	Nitrogen	26h	9.9E-04	down
D-Galactosamine	Nitrogen	24h	1.1E-04	up
D-Glucosamine	Nitrogen	24h	3.9E-04	up
g-Amino-N-Butyric Acid	Nitrogen	24h	2.8E-04	down
L-Alanine	Nitrogen	30h	6.1E-04	down
L-Pyroglutamic Acid	Nitrogen	26h	8.0E-09	down
L-Serine	Nitrogen	26h	7.2E-05	down
Nitrite	Nitrogen	30h	4.4E-05	down
Uracil	Nitrogen	30h	9.3E-04	down
Urea	Nitrogen	26h	8.3E-05	down

P-values are from Student’s Ttests comparing average growth on a given chemical for 3 replicates per isolate at a certain time point.
